# Management of Skeletal Class II Malocclusion in a Growing Patient Using Clear Aligners and Class II Elastics: A Case Report

**DOI:** 10.3390/dj14090534

**Published:** 2026-08-25

**Authors:** Francesca Gazzani, Chiara Pavoni, Francesca Chiara De Razza, Letizia Lugli, Paola Cozza, Roberta Lione

**Affiliations:** 1 Department of Medicine and Surgery, UniCamillus International Medical University, Via di Sant’Alessandro 8, 00131 Rome, Italy; francesca.gazzani@unicamillus.org (F.G.); chiara.pavoni@unicamillus.org (C.P.); lugliletizia96@gmail.com (L.L.); paola.cozza@unicamillus.org (P.C.); roberta.lione@unicamillus.org (R.L.); 2 Department of Oral and Maxillofacial Sciences, University of Rome “Sapienza”, Via Caserta 6, 00161 Rome, Italy

**Keywords:** Class II malocclusion, clear aligner treatment, Class II elastics

## Abstract

**Objective:** Class II malocclusion with mandibular retrusion in growing patients is traditionally managed with functional appliances. Recent advances in clear aligner therapy have introduced new biomechanical approaches combining aligners with intermaxillary elastics. This case report describes the correction of a skeletal Class II malocclusion using clear aligners combined with Class II elastics during the pubertal growth spurt. **Methods:** An 11-year-old female patient with skeletal Class II due to mandibular retrusion, increased overjet, deep bite, and dental Class II relationship was treated with clear aligners, transverse expansion, bite ramps, attachments, and full-time Class II elastics. **Results:** After 24 months of treatment, favorable dentoskeletal and occlusal outcomes were achieved, including improvement of the sagittal relationship (ANB reduction from 7° to 4°), mandibular advancement (SNB increase from 76° to 79°), and correction of overjet and overbite. **Conclusions:** The combination of clear aligners and Class II elastics may represent a valid treatment option for growing patients with mandibular Class II malocclusion, providing effective sagittal correction and good control of dental movements when applied during the appropriate growth phase.

## 1. Introduction

Class II malocclusions represent a complex clinical condition characterized by three-dimensional skeletal and dental disharmonies [1]. These discrepancies typically develop early during craniofacial growth and tend to persist through adolescence and young adulthood, with limited potential for spontaneous correction. The etiology of Class II malocclusion is multifactorial, involving altered muscle function, sagittal skeletal discrepancies, and interrelated changes in the transverse and vertical dimensions of the craniofacial complex [2]. The clinical management in growing patients includes a wide range of therapeutic approaches aimed to correct the underlying skeletal and dentoalveolar discrepancies. Depending on the patient’s age, growth potential, compliance, and the specific characteristics of the malocclusion, treatment options may include dentoalveolar compensation through Class II intermaxillary elastics, maxillary molar distalization protocols, mandibular advancement with functional appliances, or combinations of these strategies [3,4,5,6]. The selection of the most appropriate treatment modality is strongly influenced by the etiology and severity of the Class II relationship, the relative contribution of skeletal and dental components, the vertical growth pattern, and the stage of skeletal maturation. Consequently, an individualized treatment plan based on a comprehensive diagnostic assessment is essential to maximize treatment effectiveness and long-term stability [1,2]. Among the treatment options for growing patients showing Class II malocclusion with mandibular retrusion, functional appliances have traditionally been employed to stimulate mandibular growth and to promote forward repositioning of the mandible, improving chin projection [4]. Over time, several devices have been developed for this purpose [7,8,9,10,11], and a number of studies showed that functional appliances could induce mandibular repositioning, with the best outcomes obtained during the pubertal growth spurt [7,8,9,10,11].

In recent years, clear aligner therapy has expanded the range of available treatment options, allowing the integration of several of these biomechanical strategies [12,13].

Some authors suggested that combining intermaxillary elastics with clear aligner therapy in growing patients might produce effects comparable to those of traditional functional appliances in correcting Class II malocclusion [14,15,16]. This combination was therefore proposed as a viable alternative, offering additional advantages in terms of aesthetics and patient comfort [17].

This case report described the management of a patient with Class II malocclusion and mandibular deficiency using clear aligners in combination with Class II elastics at the growth spurt.

## 2. Materials and Methods

### 2.1. Case Presentation

The study was approved by the Ethical Committee of the Hospital of Rome “Tor Vergata” (protocol no. 48/23, 10 March 2023) and informed consent was acquired from the patient’s parents.

**Figure 1:**
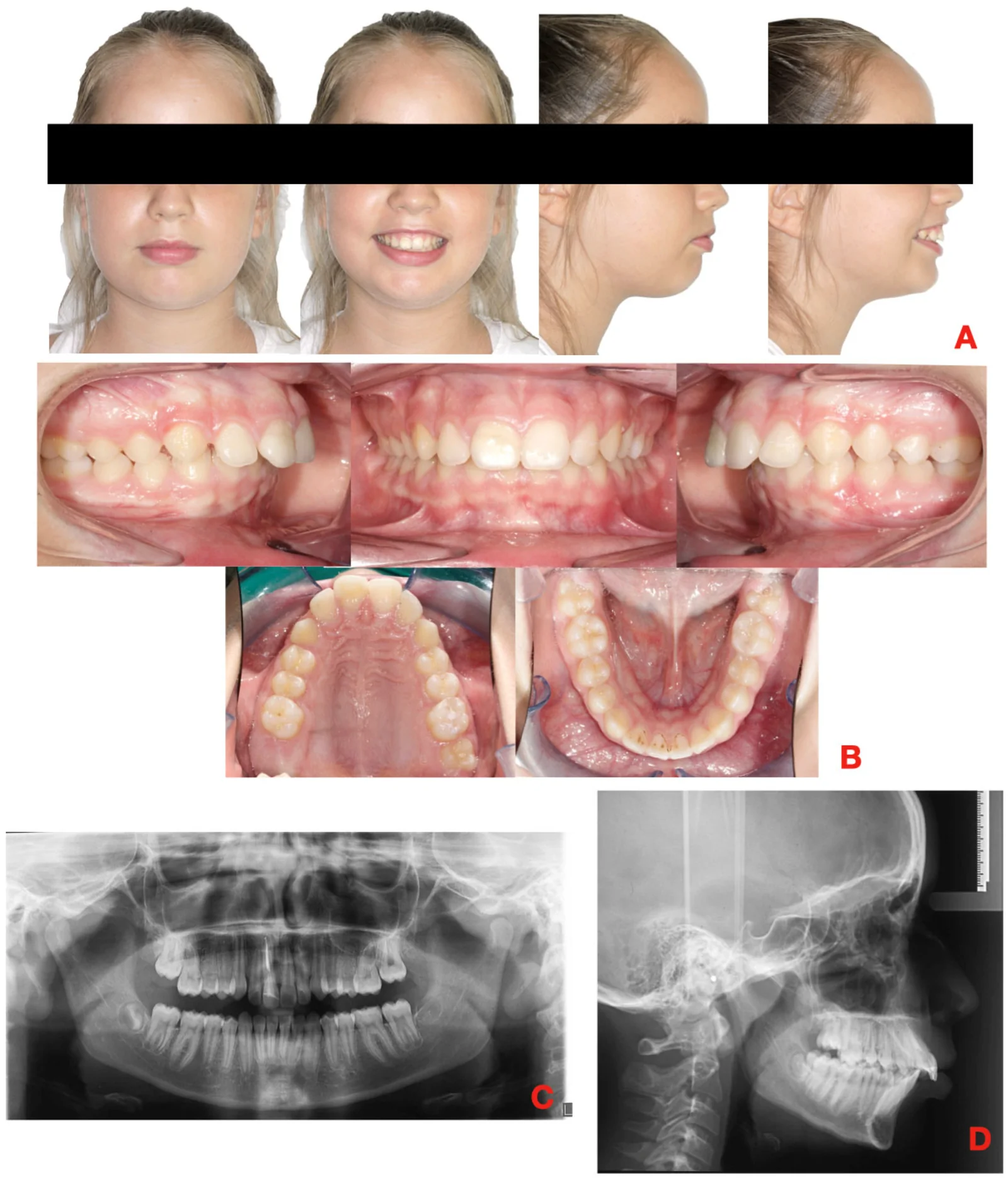
Initial records of the 11-year-old female patient at the beginning of treatment (T0). (**A**). Extra-oral photographs in frontal and lateral views. (**B**). Intra-oral photographs in frontal, lateral, and occlusal views. (**C**). Orthopantomography. (**D**). Lateral cephalogram radiograph.

An 11-year-old girl presented at clinical observation with the chief complaint of protrusive maxillary anterior teeth. Extra-oral examination (Figure 1A) revealed a symmetrical face, a convex profile, difficulty in maintaining labial seal, contraction of the perioral musculature, and a gummy smile.

Examination (Figure 1B) shows the presence of a permanent dentition, a full Class II molar and canine relationship on the left side, an end-to-end relationship on the right side with consequent deviation of the upper dental midline of 1.5 mm to the right and the lower dental midline of 1.5 mm to the left relative to the facial midline. The mandibular midline deviation was associated with the mild crowding present in the lower arch, with no clinical or radiographic evidence of mandibular asymmetry. The overjet was increased, and a severe deep bite was present, with the lower incisors contacting the cingulum area of the upper incisors. The dental arches appeared contracted, tapered, with mild crowding. Orthopantomography (Figure 1C) revealed developing lower third molar germs and the presence of a root canal treatment on maxillary right central incisor (tooth 1.1) for a previous dental trauma, with no other underlying pathologies.

As for the cephalometric analysis at T0 (Figure 1D, Table 1), a skeletal Class II relationship due to mandibular retrusion (ANB 7.0°, Wits 4 mm, SNA 83°, SNB 76°), excessive overjet (10 mm) and increased overbite (5 mm) were observed. The skeletal pattern was within normal limits (FMA 23°, SN-GoGn 37°).

Both maxillary and mandibular incisors showed an increased proinclination relative to their respective basal bones (U1 to palatal plane 118.0°, IMPA 105.0°). The skeletal maturity, as determined from the pre-treatment cephalogram using the Cervical Vertebral Maturation method, indicated a CS-3 stage [18].

A positive Frenkel maneuver, with improvement of the facial profile, was observed when the patient was asked to advance the mandible into an incisal edge-to-edge position.

The transverse dimension was assessed on T0 digital models (Table 2). Intercanine, interpremolar and intermolar widths were measured at the level of the WALA ridge on the mandibular model and at the corresponding point on the maxillary alveolar ridge. Maxillary values were 32.1 mm, 39.0 mm and 46.6 mm respectively, and mandibular values 28.3 mm, 35.8 mm and 45.5 mm. The maxillo-mandibular transverse differential at the first molars was 1.1 mm, indicating a transverse deficiency of the maxillary arch relative to the mandibular basal width.

### 2.2. Treatment Plan

A specific treatment protocol was followed, which included three key phases: transverse expansion, vertical dimension control, and sagittal correction using Class II elastics. Additional treatment objectives were levelling and aligning the arches, improving the facial profile, and achieving a more harmonious lip position.

The digital treatment plan (ClinCheck, Align Technology) was designed to combine maxillary arch expansion with distorotation of the maxillary first molars along Ricketts’ line. The planned expansion ranged from 4 to 6 mm, with an additional 2 degrees of buccal root torque applied during each expansion phase.

Direct bonded attachments were scheduled to be placed starting with the third aligner set to encourage patient compliance. Both conventional and optimized attachments were prescribed. Conventional rectangular attachments of 3 mm were used for retention and to support aligner fit, while optimized attachments were assigned by the software according to the movement required for each tooth, including rotation attachments, root control attachments and attachments supporting the transverse expansion.

In the maxillary arch, the treatment plan included arch expansion combined with retraction and intrusion of the anterior teeth to reduce overbite and enable forward repositioning of the mandible. To support overbite correction, bite ramps were incorporated on the palatal surfaces of the maxillary incisors. These bite ramps, fabricated in acrylic or resin and integrated into the aligners, temporarily disengaged the posterior occlusion and facilitated anterior intrusion. Additionally, power ridges were used to optimize torque control of the maxillary incisors during retraction.

The mandibular arch was coordinated with the maxillary arch by expanding the posterior segments and retracting the anterior teeth.

The space required for the retraction of the anterior teeth was obtained through transverse expansion of both arches, supplemented in the lower arch by interproximal reduction of 0.3 mm at each interproximal site from tooth 3.3 to tooth 4.3, performed to control the position of the mandibular incisors and to counteract the proclining effect of the intermaxillary elastics.

**Figure 2:**
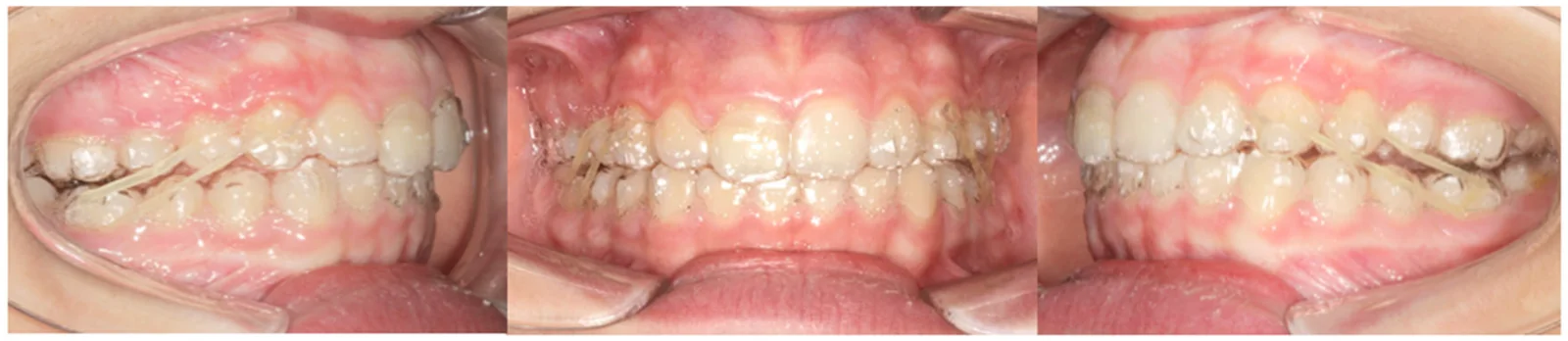
Intra-oral photographs showing the Class II elastics engaged from the precision cuts on the maxillary and mandibular aligners.

After expansion, precision cuts were designed on the aligner surface to enable the use of inter-arch elastics for sagittal correction. Class II elastics (4 oz, 3/16”) were attached from precision cuts on the maxillary canines to precision cuts in correspondence of the mandibular first molars. (Figure 2) The patient was instructed to wear the elastics full time.

Aligners were to be worn 20–22 h per day, with weekly changes. The patient was recalled every six weeks to monitor aligner fit and attachment integrity.

The patient was treated between November 2021 and November 2023.

After the active treatment, the patient started a retention protocol with Vivera retainers (Align Technology, Santa Clara, CA, USA) to be used every night for an indefinite period.

## 3. Results

Overall, the treatment lasted 24 months. The initial series involved the use of 45 aligners in the maxilla and 45 in the mandible and was completed in 11.3 months. Two refinements were subsequently carried out, of 30 and 20 aligners respectively, both with Class II elastics: the first to complete and stabilize the correction of the sagittal relationship, which had not been fully achieved within the initial series as planned in the digital setup, and the second for occlusal detailing. The transverse expansion planned in the initial setup was achieved. Attachments and precision cuts were bonded and used as planned throughout, and none required repositioning or rebonding. The elastics were worn consistently together with the aligners, as reported by the patient at the six-weekly recall appointments. The patient showed excellent compliance, and the treatment objectives were achieved.

**Figure 3:**
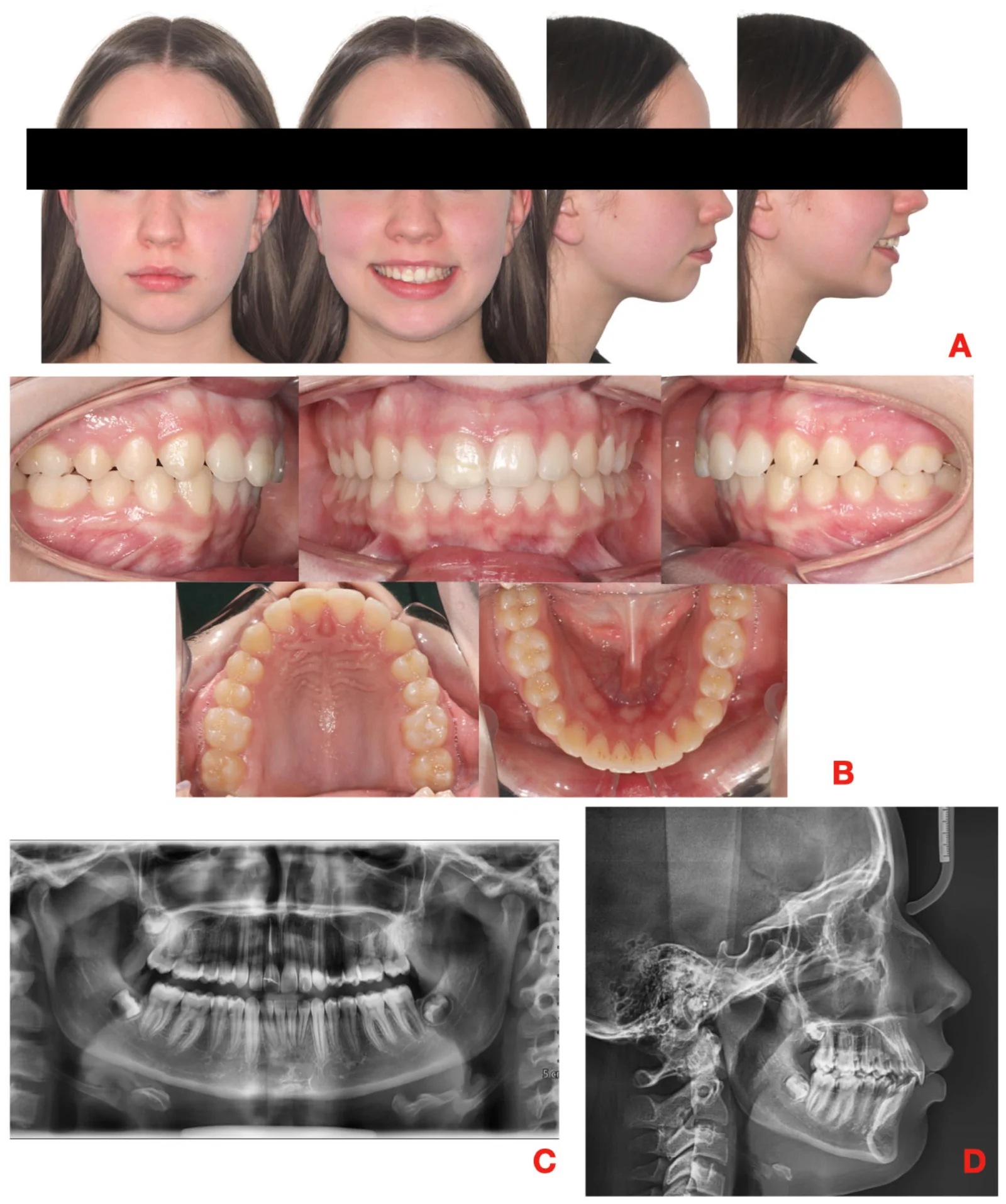
Final records of the 11-year-old female patient at the end of treatment (T1). (**A**). Extra-oral photographs in frontal and lateral views. (**B**). Intra-oral photographs in frontal, lateral, and occlusal views. (**C**). Orthopantomography. (**D**). Lateral cephalogram radiograph.

At the end of treatment (T1), extra-oral analysis (Figure 3A) revealed an improvement in the soft tissue profile and proper incisor exposure.

The transverse dimension was reassessed on the T1 digital models (Table 2). Maxillary widths increased from 32.1 to 33.6 mm (intercanine), from 39.0 to 41.1 mm (interpremolar) and from 46.6 to 48.9 mm (intermolar), whereas the mandibular values were substantially unchanged (28.3 to 28.7 mm, 35.8 to 36.3 mm and 45.5 to 46.0 mm). The maxillo-mandibular transverse differential at the first molars increased from 1.1 mm to 2.9 mm, the change in inter-arch coordination being accounted for almost entirely by the maxillary arch.

Moreover, intra-oral examination (Figure 3B) revealed symmetrical and well-shaped arch forms, the achievement of a bilateral Class I molar and canine relationship, and the recovery of correct overbite and overjet values. The post-treatment orthopantomography and lateral cephalometric radiograph are shown in Figure 3C,D.

**Figure 4:**
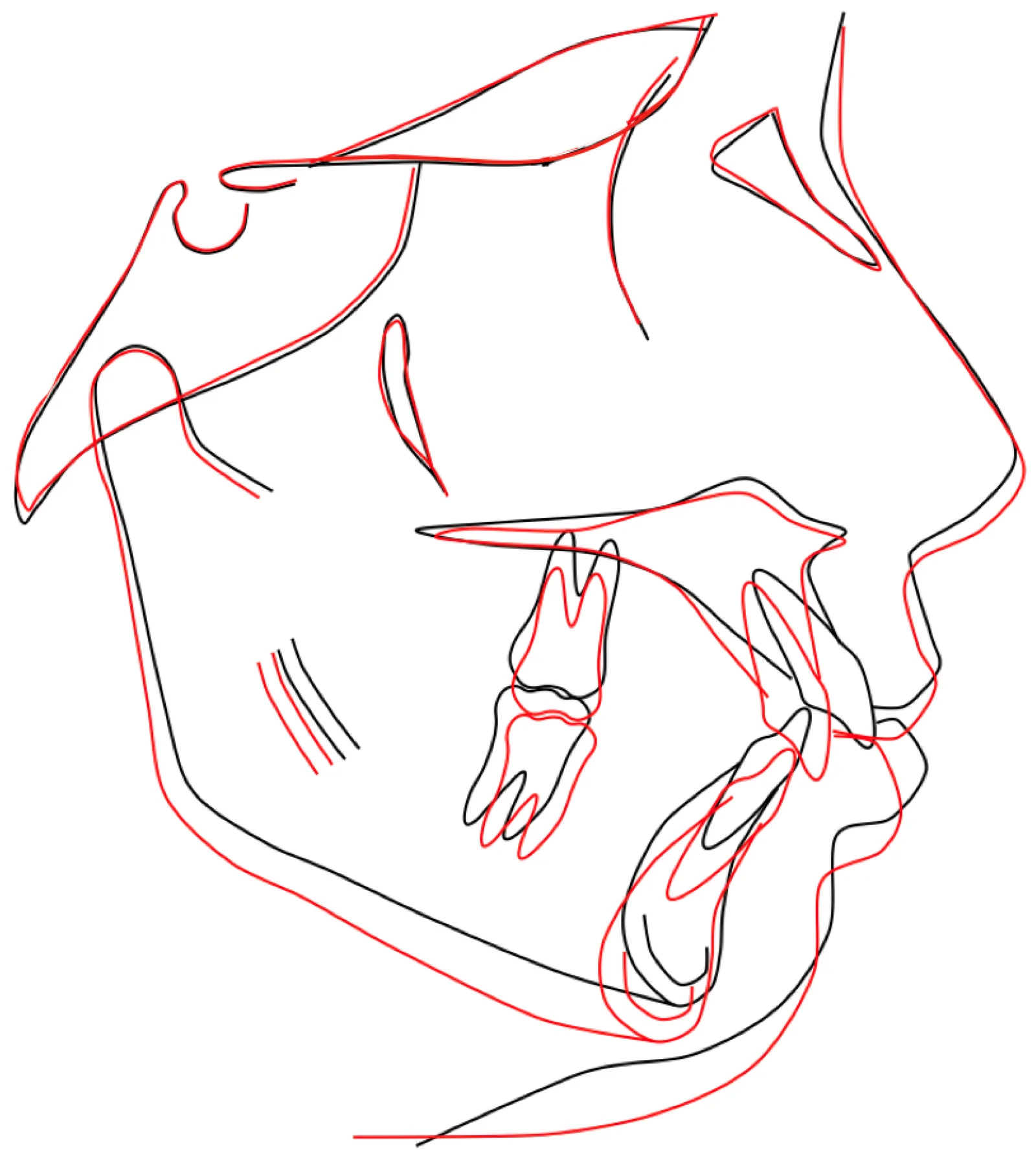
Structural superimposition of the T0 (black) and T1 (red) cephalometric tracings on the anterior cranial base, according to Björk’s method.

The effects of therapy were also evaluated on lateral cephalogram (Table 1) at the end of treatment (T1). In particular, the ANB angle decreased by 3° (from 7° at T0 to 4° at T1), while SNA remained unchanged (83°) and SNB increased from 76° at T0 to 79° at T1. Structural superimposition of the T0 and T1 tracings on the anterior cranial base (Figure 4) showed an anterior and inferior displacement of the mandible, with pogonion and menton repositioned forward and downward, while the maxilla remained unchanged in the sagittal plane. The reduction in the sagittal intermaxillary discrepancy was therefore accounted for by the change in mandibular position relative to the cranial base. As the patient was treated through the pubertal interval, having been at CS3 at T0, and no untreated control was available, the respective contributions of the treatment mechanics and of the mandibular growth expected over the same period cannot be precisely separated. Both mandibular and maxillary incisor proclination were reduced, with a more pronounced change observed in the mandibular incisors (IMPA: 105° T0, 97° T1; U1/PF: 118° T0, 105° T1). In addition, a correction of overjet and overbite was achieved, decreasing from 10 mm to 2 mm and from 5 mm to 2.5 mm, respectively).

The soft tissue profile was analysed on the T0 and T1 lateral cephalograms using the true vertical line (TVL) as reference. Soft tissue A point was essentially unchanged (−0.9 mm at T0, −0.7 mm at T1), whereas soft tissue B point advanced from −11.0 mm to −7.8 mm and soft tissue pogonion from −12.2 mm to −7.6 mm. Upper lip anterior moved from 2.1 mm to 3.1 mm and lower lip anterior from 0.2 mm to 0.6 mm; the upper incisor tip was retracted from −6.6 mm to −9.6 mm and the lower incisor tip moved from −14.0 mm to −13.1 mm. Lip seal was obtained without perioral muscular strain and incisor exposure at rest was reduced. At T1 soft tissue pogonion remained posterior to the TVL, consistent with a residual Class II soft tissue pattern.

## 4. Discussion

Clear aligners combined with Class II elastics have been described in growing patients as a means of addressing Class II malocclusion, and studies comparing this approach with functional appliances have been published [17,18,19,20]. The present report describes the clinical course of a single patient treated with this protocol; the design does not allow any comparison with alternative treatment modalities, nor any inference about the mechanism through which the occlusal correction was obtained.

A structured treatment sequence was followed, consisting of transverse arch expansion, vertical dimension control, and sagittal correction through Class II elastics. Expansion of the transverse dimension helps reduce of the occlusal interferences enabling more controlled and coordinated antero-posterior movements. Given that up to 85% of Class II patients exhibit mesial rotation of the maxillary first molars [3,20,21], rotational correction was also incorporated during the expansion phase to improve inter-arch coordination.

Inter-arch mechanics with Class II elastics were used to recover the correct sagittal relationship, promoting functional rebalancing of the oral and perioral musculature. However, the application of Class II elastics may create constrictive forces on the maxillary arch, which could partially limit the programmed expansion observed in the digital setup (ClinCheck) [22]. Based on the authors’ experience, the use of Class II elastics is suggested after the transverse expansion phase has been completed.

To enhance aligner retention and optimize the effectiveness of inter-arch mechanics, customized composite resin attachments were employed. Additionally, precision cuts were strategically designed to control incisor proclination: a hook was incorporated in the maxillary aligner to avoid further proclination of the upper incisors, while a buccal button on the mandibular first molar was positioned to counteract unwanted lower incisor proclination.

Several studies have reported undesirable side effects of Class II elastics, including mandibular anchorage loss, mandibular molar extrusion, clockwise rotation of the occlusal plane, and proclination of the mandibular incisors [20]. In the present patient the vertical variables showed a slight increase between T0 (FMA 23°; SN/Go-Me 37°) and T1 (FMA 25°; SN/Go-Me 38°). A key benefit of clear aligners lies in their bite-block effect, which effectively preserve the vertical dimension by minimizing posterior dental extrusion, despite the use of Class II elastics that might otherwise promote vertical side effects. Moreover, applying Class II elastics with aligners may provide greater freedom of mandibular movement, potentially facilitating mesial mandibular repositioning during the pubertal growth spurt.

The transverse changes recorded between T0 and T1 are to be regarded as dentoalveolar in nature: no skeletal expansion device was used, and the aligners act on the alveolar processes rather than on the midpalatal suture. The stability of the mandibular basal widths, which changed by less than 0.5 mm, supports this interpretation for the lower arch, whereas the maxillary arch accounted for almost the entire improvement in inter-arch coordination. In a patient aged 11 years at the start of treatment, a contribution of the residual transverse growth of the maxilla cannot be excluded and cannot be quantified from the present records.

Finally, it should be emphasized that treatment success depends strongly on patient compliance with aligner wear. If compliance is suboptimal, the therapeutic goals may not be fully achieved. Therefore, accurate case selection is essential, considering the increased need for patient cooperation when using elastics. Nevertheless, the comfort and aesthetic advantages offered by clear aligners are likely to promote high compliance, making them a promising treatment option for appropriately selected growing patients.

This case report has inherent limitations related to its single-case design, which does not allow generalization of the results to a broader population of growing patients with Class II malocclusion. The absence of a control group further limits the ability to attribute the observed skeletal and dental changes solely to the described treatment protocol, since some degree of spontaneous mandibular growth cannot be excluded during the pubertal growth spurt. Additionally, the relatively short observation period does not provide information regarding the long-term stability of the sagittal correction achieved, and relapse following the removal of the aligners and elastics cannot be ruled out.

Future studies involving larger samples and prospective controlled designs are needed to confirm the effectiveness of clear aligners combined with Class II. Long-term follow-up studies are also warranted to assess the stability of the dentoskeletal outcomes over time. Comparative studies directly evaluating this approach against traditional functional appliances within the same population would further help define the specific indications, limitations, and predictability of this treatment modality.

## 5. Conclusions

In the patient described here, a protocol combining clear aligners with Class II elastics was associated with the correction of the molar and canine relationship and with a reduction of overjet and overbite over 24 months of active treatment. The report illustrates the clinical application of intermaxillary Class II elastics within an aligner-based protocol in a patient treated at the pubertal growth peak, and documents the mechanics employed and the outcome obtained in this individual case. These observations relate to a single case treated without a control group and do not permit conclusions regarding the efficacy, the predictability or the reproducibility of this approach, nor its performance relative to other treatment modalities. Controlled studies with an adequate sample size and post-retention follow-up are required before the role of this protocol in growing patients can be established.

## Figures and Tables

**Table 1:** Cephalometric comparison of T0-T1 changes.

Variables	Norm	T0	T1
Sagittal skeletal			
SNA	82 ± 2°	83°	83°
SNB	80 ± 2°	76°	79°
ANB	2 ± 2 °	7°	4°
WITS	0 ± 2 mm	4 mm	2 mm
Vertical skeletal			
FMA	25 ± 3°	23°	25°
SN/Go-Me	33 ± 5°	37°	38°
SN/ANS-PNS	8 ± 3°	8°	8°
ArGoMe	130 ± 7°	124°	124°
Dento-basal			
Maxillary incisor inclination (U1^PF)	105–110°	118°	105°
Mandibular incisor inclination (IMPA)	94 ± 5°	105°	97°
Overjet	2.5 ± 2.5 mm	10 mm	2 mm
Overbite	2.5 ± 2.5 mm	5 mm	2.5 mm
Soft tissue analysis			
Soft tissue A point (A’-TVL)	−0.1 ± 1.0	−0.9 mm	−0.7 mm
Upper lip anterior (ULA-TVL)	3.7 ± 1.2	2.1 mm	3.1 mm
Upper incisor tip (Mx1-TVL)	−9.2 ± 2.2	−6.6 mm	−9.6 mm
Lower incisor tip (Md1-TVL)	−12.4 ± 2.2	−14 mm	−13.1 mm
Lower lip anterior (LLA-TVL)	1.9 ± 1.4	0.2 mm	0.6 mm
Soft tissue B point (B’-TVL)	−5.3 ± 1.5	−11 mm	−7.8 mm
Soft tissue Pogonion (Pog’-TVL)	−2.6 ± 1.9	−12.2 mm	−7.6 mm

**Table 2:** T0-T1 transversal changes.

Transverse (WALA Ridge)		
Maxillary intercanine width	32.1 mm	33.6 mm
Maxillary interpremolar width	39 mm	41.1 mm
Maxillary intermolar width	46.6 mm	48.9 mm
Mandibular intercanine width	28.3 mm	28.7 mm
Mandibular interpremolar width	35.8 mm	36.3 mm
Mandibular intermolar width	45.5 mm	46 mm
Maxillo-mandibular differential (molars)	1.1 mm	2.9 mm

## Data Availability

The data supporting the findings of this study are available from the corresponding author upon reasonable request. Due to privacy and ethical restrictions, the clinical data and images cannot be publicly shared.
